# Potential influence of selection criteria on the demographic composition of students in an Australian medical school

**DOI:** 10.1186/1472-6920-11-97

**Published:** 2011-11-23

**Authors:** Ian B Puddey, Annette Mercer, Sandra E Carr, William Louden

**Affiliations:** 1 Faculty of Medicine, Dentistry and Health Sciences, University of Western Australia, 35 Stirling Hwy, Crawley, WA 6009, Australia; 2 Vice-Chancellery, University of Western Australia, 35 Stirling Hwy, Crawley, WA 6009, Australia

## Abstract

**Background:**

Prior to 1999 students entering our MBBS course were selected on academic performance alone. We have now evaluated the impact on the demographics of subsequent cohorts of our standard entry students (those entering directly from high school) of the addition to the selection process of an aptitude test (UMAT), a highly structured interview and a rural incentive program.

**Methods:**

Students entering from 1985 to 1998, selected on academic performance alone (N = 1402), were compared to those from 1999 to 2011, selected on the basis of a combination of academic performance, interview score, and UMAT score together with the progressive introduction of a rural special entry pathway (N = 1437).

**Results:**

Males decreased from 57% to 45% of the cohort, students of NE or SE Asian origin decreased from 30% to 13%, students born in Oceania increased from 52% to 69%, students of rural origin from 5% to 21% and those from independent high schools from 56% to 66%. The proportion of students from high schools with relative socio-educational disadvantage remained unchanged at approximately 10%. The changes reflect in part increasing numbers of female and independent high school applicants and the increasing rural quota. However, they were also associated with higher interview scores in females vs males and lower interview scores in those of NE and SE Asian origin compared to those born in Oceania or the UK. Total UMAT scores were unrelated to gender or region of origin.

**Conclusions:**

The revised selection processes had no impact on student representation from schools with relative socio-educational disadvantage. However, the introduction of special entry quotas for students of rural origin and a structured interview, but not an aptitude test, were associated with a change in gender balance and ethnicity of students in an Australian undergraduate MBBS course.

## Background

In 1999 MBBS students entering the University of Western Australia (UWA) Medical School did so via a revised selection process which included the score from a highly structured interview [1] and the score on an aptitude test-the Undergraduate Medicine and Health Sciences Admission Test (UMAT) [1], in addition to the previous single entry criterion of academic performance. The main goals of the revised selection process were to increase the diversity in the student cohorts, enhance equity in the selection of students and to select students who would be highly motivated to become doctors, with an anticipated lowering of the relatively high attrition rate evident in the course at that time.

The changes in the selection processes were paralleled by the progressive imposition of special entry quotas for rural students in compliance with the parameters of the Rural Undergraduate Support and Coordination Program (Department of Health & Ageing, Commonwealth Government of Australia) and on a substantially smaller scale, via other University of Western Australia defined pathways established specifically for students with socio-educational or other disadvantage (UWay and Outer Urban pathways).

Subsequently we have seen major shifts in the demographic composition of standard entry students entering our MBBS course with respect to gender, origin from rural vs metropolitan regions, country of origin, high school background (public vs independent) and socio-educational advantage. Some of these changes have resulted directly from the special entry quotas and others through changes in the broader demographics of the Western Australian population. This study evaluated the possible further influence of either the structured interview or UMAT, and analysed the relationship of each of the selection components with the demographic composition of student cohorts both before and after commencement of the revised selection process.

## Methods

The study population comprised all standard entry students from 1985 to 2011 (students directly from high school with no prior tertiary study) (N = 2839). They were divided into 2 cohorts-those prior to 1999 who were selected on the basis of academic performance alone (N = 1402) and those after 1999 selected on the basis of a combination of academic performance, the score from a highly structured interview and the score on an aptitude test-the UMAT (N = 1437) [1]. Academic performance before 1998 was assessed by a Tertiary Entry Score (TES) calculated from the best of 3 to 5 subjects, with a maximum score of 510. From 1999 onwards it was assessed by Tertiary Entrance Rank (TER) (maximum score 99.95) which is calculated on the basis of the total number in the cohort and the TES distribution for that year. All analyses were conducted with the inclusion of those students selected through the special entry quota pathways which comprised 40 students prior to 1999 (2.9%) and 276 students from 1999 onwards (19.2%).

The basic format and components of the highly structured interview developed at UWA have been reported previously [2]. In 2007 a change in the interview scoring was instituted so that each of the 7 components of the interview score was ranked on a 6 point score rather than a 4 point score. The total and component interview scores have therefore been analysed with all values from 2007 standardised to a 28 point scale rather than the raw interview score. Only 2 of the 7 components have been consistently assessed since 1999-the global communication skills score and the motivation/commitment score-and so sub-analyses are also presented against the standardised score for each of these components.

Even though the total UMAT score alone was used in the ranking process, each of the three component scores, UMAT1 (Logical reasoning and problem solving), UMAT2 (Understanding people) and UMAT3 (Non-verbal reasoning) have different and independent constructs [3] and have therefore been independently evaluated in this study together with the total score.

An Index of Community Socio-Educational Advantage (ICSEA) has now been developed for all schools in Australia [4] and is listed for each school on the MySchool website http://www.myschool.edu.au/. In this study, in an attempt to discern any changes in student socio-economic background pre and post introduction of the new admission process, we have imputed for all Western Australian students an ICSEA score on the basis of the high school in which they completed their TES or TER. The score is calculated from a number of variables which include parental occupation, parental school education level, parental non-school education level, percentage of families that are one parent families with dependent offspring only, percentage of occupied private dwellings with no internet connection, percentage of Aboriginal enrolments, an accessibility/remoteness index, and the percentage of students with both a language background other than English and parents with an education level of Year 9 equivalent or below. Every school has an ICSEA value on a scale which has a mean within Australia of 1000 and a standard deviation of 100. ICSEA values range from around 500 (representing extremely disadvantaged backgrounds) to about 1300 (representing schools with students from very advantaged backgrounds). The value on the scale assigned to each school is the averaged level for all students in that particular school. School of origin (and therefore ICSEA score) was unavailable for approximately 9% of the cohort.

Region of origin was determined from country of origin according to major regional groups as outlined in the Australian Standard Classification of Countries for Social Statistics [5]. Data were available for 2821 students. Given the relatively small numbers of students in some groups they have been collapsed into 5 groups for analysis-those from Oceania (Australia, New Zealand, Papua New Guinea and proximate Pacific islands), UK and Ireland, NE and SE Asia, Southern Asia (India, Pakistan, Sri Lanka and Bangladesh) and Other.

### Statistics

Univariate comparisons of each demographic characteristic or each selection criteria utilised either independent sample T-tests, chi square analysis or one-way analysis of variance (with post-hoc comparisons by Bonferroni correction), as appropriate. Multivariate analyses utilised generalised linear modelling (GLM) to assess the main effects of gender, high school of origin, region of origin or special entry pathway status entered as predictive factors, ICSEA score entered as a predictive covariate and TER, TES, UMAT or interview scores (or their component parts) entered as dependent variables. All analyses were carried out utilising Predictive Analytics SoftWare Statistics Release 18.0.1, 2009.

## Results

### Demographic Characteristics

#### Gender

**Figure 1:**
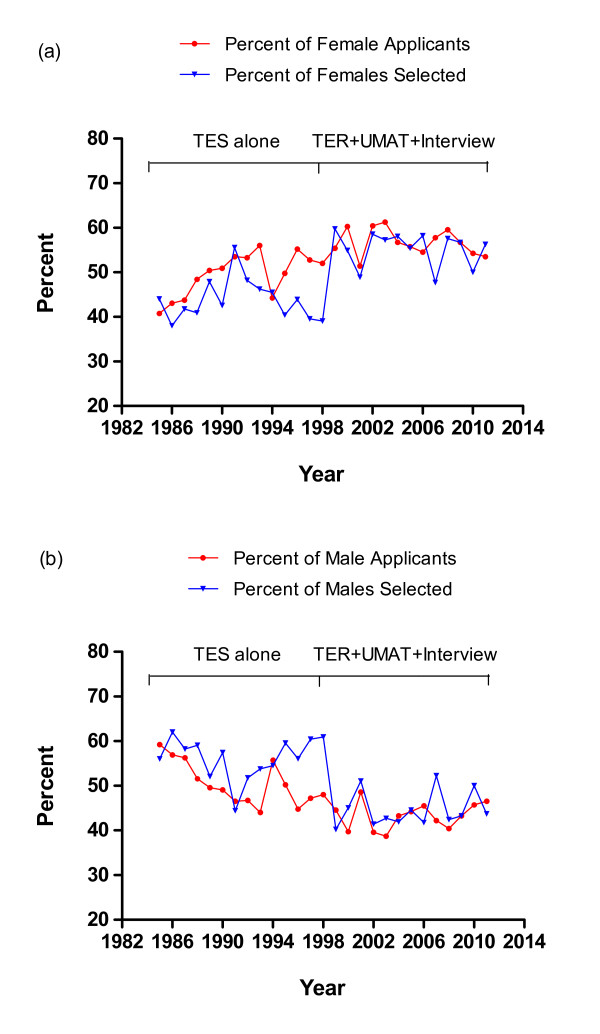
**1a and 1b: Gender of standard entry students to MBBS course from WA high schools 1985 to 2011 (Total number of applicants N = 7935, Total selected N = 2594)**.

Males were predominant prior to the introduction of the revised selection process (males 56.8%-N = 794, females 43.2%-N = 604) and females after (males 45.7%-N = 659, females 54.3%-N = 782) (Chi-squared = 34.8, P < 0.001). For all applicants from WA high schools from 1985 to 2011 (N = 7935) a further analysis was undertaken to compare the relative proportion by gender of those selected as against the relative proportion who applied. From 1985 to 1998, males comprised 50.4% of the applicants, but represented 56.2% of those selected to the course (Figure 1a). This was nearly completely reversed after the introduction of the revised selection process. From 1999 to 2011 the proportion of female applicants increased to 56.7% and the proportion of females selected to 55.5% (Figure 1b).

#### High School Location and Type and Socio-Educational Disadvantage

**Figure 2:**
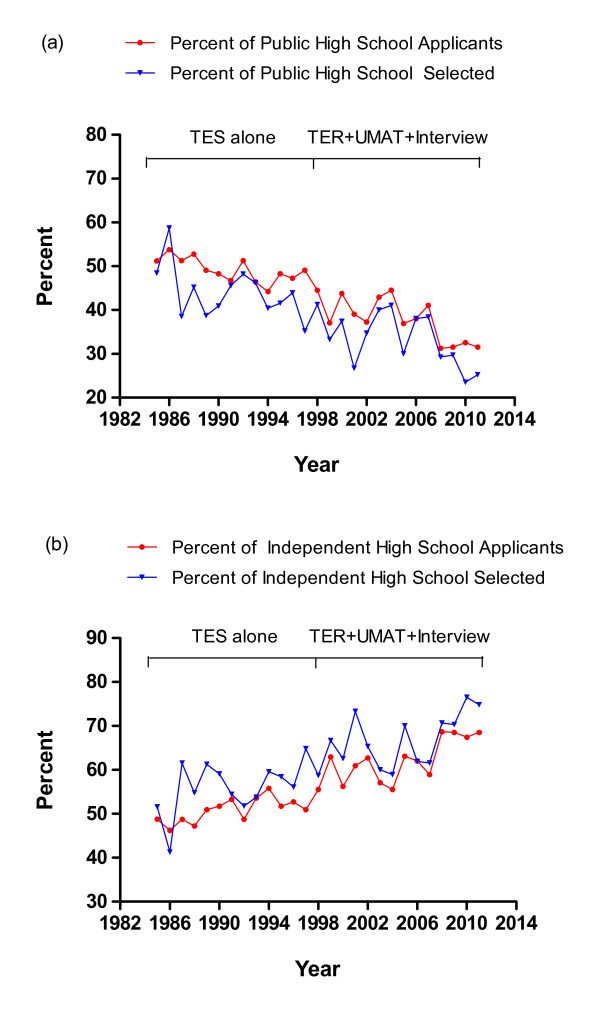
**2a and 2b: Percentage of independent vs public high school WA TEE applicants and selected students for the MBBS course 1985 to 2011 (WA students only, N = 2594)**.

The proportion of applicants from Western Australian (WA) public (government-funded) high schools (N = 7935), who listed the MBBS course as their first preference, fell steadily from a high of 52% during the 5-year period 1985 to 1989 to 33% during the five years 2007 to 2011. Successful applicants from public high schools fell equally steadily from a high of 46% during the period 1985 to 1989 (when approximately 72% of all students attended public high schools) ^6 ^to 29% during the period 2007 to 2011 (when approximately 59% of all students attended public high schools) [6] (Figure 2a). Conversely, during the same intervals the proportion of applicants from WA independent (fee paying) high schools, who listed the MBBS course as their first preference, increased steadily from 48% to 67% while the proportion of students actually admitted to the course from independent high schools increased from 54% to 71%, with the overall proportion of all students enrolled in independent high schools increasing from approximately 25% to 41% over the same period [6] (Figure 2b).

**Figure 3:**
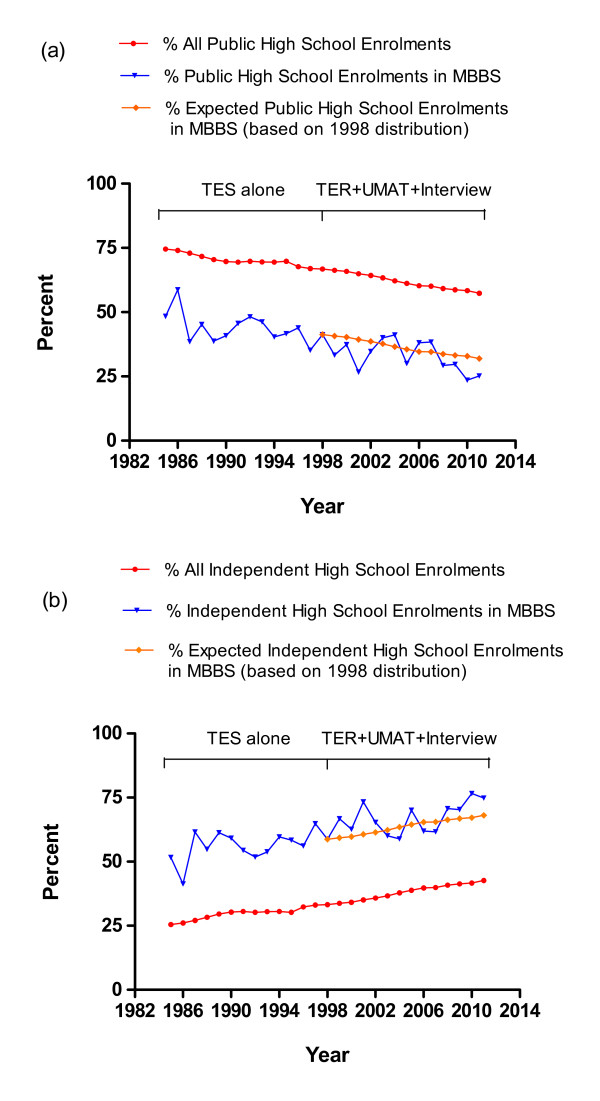
**3a and 3b: Percentage of independent vs public high school students in WA and percentage of independent vs public high school students of standard entry students to MBBS course 1985 to 2011 (WA students only, N = 2594, actual vs expected)**.

Based on public and independent high school enrolments from 1999 to 2011 [6], and using the distribution between independent and public school admissions to the MBBS course in 1998, the predicted distribution for MBBS admissions (if no change had been made to the selection processes) was calculated. A subsequent comparison of the actual vs predicted distribution between independent and public school admissions from 1999 to 2011 suggested no substantial influence of the revised selection process on the relatively lower proportion of admissions from public vs independent high schools than that expected in the face of declining public high school enrolments alone (Figure 3a and 3b).

A further analysis was undertaken utilising the ICSEA score for the school of origin for each student. ICSEA scores were significantly higher in those selected from independent high schools compared to public high schools (1150.1 ± 1.4 vs 1071.6 ± 2.5, P < 0.001, respectively). The proportion of students selected from high schools with an ICSEA score ≤ 1000 before and after the revised selection process was 10.9% and 9.3% respectively. No change in the mean ISCEA score of the school of origin of successful students was seen when comparing the period before the revised selection processes began to that after (1117.8 ± 2.1 vs 1120.9 ± 2.0; NS). However, if students selected from public high schools were analysed in isolation, a significant increase was seen (1086.6 ± 3.3 to 1102.2 ± 2.9; P < 0.001, for public metropolitan schools and 949.2 ± 5.0 to 962.6 ± 3.3, P = 0.023, for public rural schools) with no significant change seen for metropolitan or rural independent high schools.

#### Region of Origin

**Figure 4:**
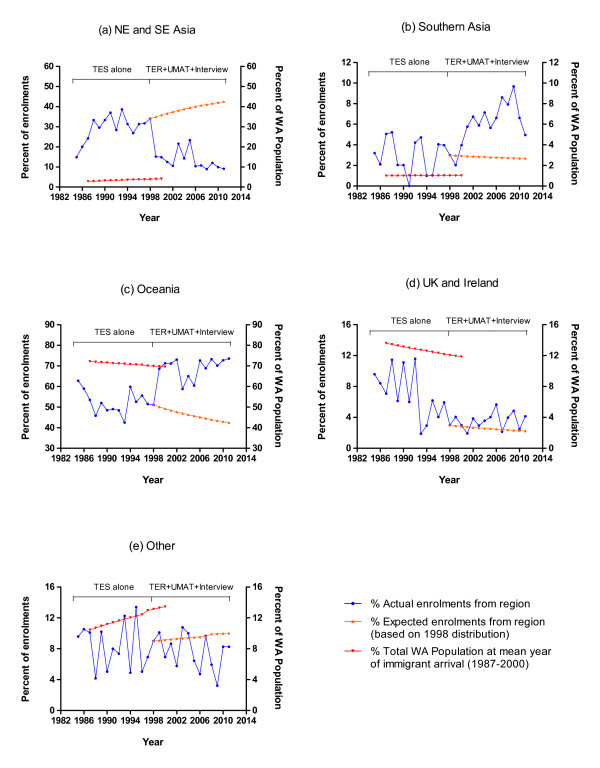
**Region of origin of high school student enrolments to MBBS course 1985 to 2011 (N = 2821)**.

During the period when entry was on the basis of academic performance alone, the proportion of students from a NE or SE Asian country of origin increased dramatically from 15% in 1985 to 34% by 1998. This was on a background of NE or SE Asian immigrants constituting only 3-4% of the population of WA [7]. After the introduction of the revised selection process, the proportion of students from an NE or SE Asian country of origin fell equally dramatically to 15% in 1999 and to 9% by 2011 (Figure 4a). The mean period since arrival in Australia for all students admitted after 1999 and with a region of origin apart from Oceania was 11.2 ± 0.2 years. Therefore the anticipated percentage of successful NE and SE Asian applicants for the course if there had been no change to the selection process was calculated on the basis of the relative NE or SE Asian region of origin distribution in the 1998 cohort together with the relative proportion of the WA population from a NE or SE Asian country of origin 12 years earlier (1987 to 2000) [7]. This indicated a mean expected percentage of 39% from 1999 to 2011 vs the actual recorded mean of 13% (Figure 4a).

In contrast, students from a Southern Asian origin remained at a base proportion of approximately 3% from 1985 to 1998 but more than doubled over the period 1999 to 2011 (Figure 4b). This was on a background of Southern Asian immigrants constituting only 1% of the total population of WA [7]. The mean anticipated percentage from 1999 to 2011 would have been 2.8% vs the actual recorded mean of 6.3% (Figure 4b). Students born in Oceania increased from an approximate mean of 52% of the successful cohort from 1985 to 1998 to 69% from 1999 to 2011, much more closely approximating their 70% proportion of the total WA population (Figure 4c) [7]. If the selection process had remained unchanged the mean expected percentage from 1999 to 2011 would have been 46% (Figure 4c) [7]. Students from UK and Ireland combined were generally selected in a lower proportion relative to their decreasing percentage of the population of WA, a pattern which was for the most part unchanged after the revised selection processes with the mean anticipated percentage from 1999 to 2011 being 2.3% vs an actual recorded mean of 3.6% (Figure 4d). Students from all Other regions of origin were generally selected in a lower proportion relative to their increasing percentage of the population of WA, with no change in this pattern following the introduction of the revised selection processes. The mean anticipated percentage from 1999 to 2011 was 9.5% vs an actual recorded mean of 7.6% (Figure 4e).

#### Rural vs Metropolitan Origin

From a low of 2.8% from 1985 to 1990 the proportion of students of rural origin increased to 26.9% during 2007 to 2011. This reflected an active recruitment process for the rural special entry pathway over this period with 54% of students of rural origin admitted via the rural special entry pathway up to 1998 and 82% admitted via the rural special entry pathway after 1999. However, it also reflected changing definitions of "rural and remote origin" over the period as well as better ascertainment of those with a rural background. Recruiting one in four students with a rural origin was linked to other demographic changes in the medical student cohort. Up to 1998, 75% of the students with a rural origin graduated from a public high school compared to 43% of those of non-rural origin. After 1999, as the rural quota increased, the number of students with a rural origin who had graduated from a public high school fell to 41% compared to a fall to 32% of those who were of a non-rural origin (Chi-squared = 26.6, P < 0.001). Both up to 1998 and from 1999 onwards, the rural origin cohort remained steadily of predominantly Oceanic region of origin (86% of the cohort). Very few rural origin students were of NE or SE Asia region of origin (approximately 3%) and so a substantial component of the fall in NE or SE Asia region of origin students selected for the course was a direct result of the gradual imposition of a 25% rural quota. Up to 1998 the proportion of students of rural origin who were male and female was similar and so the increasing feminisation of the student cohort after 1999 tended to be less in students of rural origin (Chi-squared = 2.4, NS).

### Selection Parameters

#### Academic Performance

##### Entrants Before 1999 - Tertiary Entrance Score

Univariate associations of demographic characteristics with the TES are listed in Table 1. The ICSEA score was correlated positively with the TES (r = 0.22, P < 0.001). Multivariate analysis (GLM) of all students from WA metropolitan and rural high schools who entered from 1985 to 1998 (N = 1261) (interstate and New Zealand entrants or any case with missing data excluded) revealed significant model effects on TES for gender (P < 0.001), high school type (P = 0.002) and selection via special entry pathways (P < 0.001), but not region of origin. Parameter estimates indicated that TES was 5.1 ± 1.1 higher in males compared to females (P < 0.001). Compared to students from independent metropolitan high schools, TES was 10.4 ± 3.7 lower in those from rural public high schools (P = 0.004) and 3.2 ± 1.1 lower (P = 0.004) in those from metropolitan public high schools (P < 0.001). Compared to standard pathway entrants, TES in those who entered via UWay was 59.6 ± 8.7 lower (P < 0.001) and rural special entry was 39.5 ± 4.7 lower (P < 0.001). If the ICSEA score was included as a covariate in the model, rather than the factor, high school type, there was a significant model effect (P = 0.001) with higher TES predicted for schools with a higher ICSEA score.

**Table 1:** Academic score at entry vs student demographic characteristics prior to the revised selection processes (1985 to 1998).

	TES	P Value (T-test)
Gender		
Male (N = 760)	457.9 ± 1.0	P < 0.001
Female (N = 579)	452.4 ± 1.2	

High School		
Independent High School (N = 709)	459.2 ± 0.8	P < 0.001
Public High School (N = 567)	454.1 ± 0.9	

Special Entry Pathways		
Special Entry (N = 37)	408.9 ± 2.6	P < 0.001
No Special Entry (N = 1302)	456.8 ± 0.8	

Region of Origin		
Oceania (N = 696)	454.3 ± 1.1	
UK & Ireland (N = 88)	458.7 ± 2.0	NS
NE & SE Asia (N = 392)	458.7 ± 1.0	
Southern Asia (N = 36)	454.2 ± 3.0	
Other (N = 112)	450.3 ± 5.0	

TES-Tertiary Entrance Score.

##### Entrants Since 1999-Tertiary Entrance Rank

Univariate associations of demographic characteristics with the TER are listed in Table 2. The ICSEA score was correlated positively with the TER (r = 0.28, P < 0.001). Multivariate analysis (GLM) of all students from WA metropolitan and rural high schools who entered from 1999 to 2011 (N = 1294) (interstate and New Zealand entrants or any case with missing data excluded) revealed significant model effects on TER for gender (P < 0.001), high school type (P < 0.001), region of origin (P < 0.001) and selection via special entry pathways (P < 0.001). Parameter estimates indicated that TER was 0.21 ± 0.06 higher in males compared to females (P < 0.001). Compared to students from independent metropolitan high schools, TER was 0.49 ± 0.13 lower in those from rural public high schools (P < 0.001), 0.40 ± 0.13 lower in those from rural independent high schools (P = 0.001) and 0.16 ± 0.07 lower (P = 0.023) in those from metropolitan public high schools (P < 0.001). Compared to students born in Oceania it was 0.28 ± 0.09 higher in those from NE and SE Asia (P = 0.001) and 0.45 ± 0.15 lower in those from the UK and Ireland (P = 0.003). Compared to standard pathway entrants TER in those who entered via UWay was 2.46 ± 0.31 lower (P < 0.001), rural special entry was 0.92 ± 0.10 lower (P < 0.001) and outer urban special entry was 0.84 ± 0.23 lower (P < 0.001). If the ICSEA score was included as a covariate in the model, rather than the factor, high school type, there was a significant model effect (P = 0.001) with higher TER predicted for schools with a higher ICSEA score.

**Table 2:** Academic score at entry vs student demographic characteristics after the revised selection processes (1999 to 2011).

	TER	P Value (T-test or ANOVA)
Gender		
Male (N = 657)	98.88 ± 0.04	P < 0.001
Female (N = 780)	98.64 ± 0.04	

High School		
Independent High School (N = 856)	98.86 ± 0.04	0.013
Public High School (N = 439)	98.68 ± 0.06	

Special Entry Pathways		
Special Entry (N = 276)	97.71 ± 0.09	P < 0.001
No Special Entry (N = 1161)	99.00 ± 0.03	

Region of Origin		
Oceania (N = 993)	98.65 ± 0.04	
UK & Ireland (N = 52)	98.36 ± 0.15	P < 0.001
NE & SE Asia (N = 193)**	99.19 ± 0.06	
Southern Asia (N = 91)* ^#^	99.03 ± 0.09	
Other (N = 107)	98.86 ± 0.10	

ANOVA post hoc comparisons with Bonferroni correction-* P < 0.05, ** P < 0.001 vs Oceania, ^# ^P < 0.01, ^## ^P < 0.001 vs UK & Ireland. TER-Tertiary Entrance Rank.

#### UMAT Score

##### UMAT1 Score (Logical reasoning and problem solving)

Univariate associations of demographic characteristics with UMAT1 score are listed in Table 3. The score for UMAT1 was lower in females compared to males. It was also lower in students of Eastern and SE Asian origin compared to both those born in Oceania or of UK & Irish origin and lower in students from public high schools compared to those from independent schools. Multivariate analysis (GLM) confirmed significant model effects on UMAT1 for gender (P < 0.001), region of origin (P < 0.001) and selection via special entry pathways (P < 0.001). Parameter estimates indicated that UMAT1 was 2.2 ± 0.4 higher in males compared to females (P < 0.001). There was no significant model effect from high school of origin. Compared to students born in Oceania, UMAT1 was 3.4 ± 0.7 lower in those from NE and SE Asia (P = 0.001) and 2.8 ± 0.9 lower in those from Southern Asia (P = 0.003). Compared to standard pathway entrants UMAT1 in those who entered via UWay was 10.2 ± 2.4 lower (P < 0.001) and rural special entry was 3.8 ± 0.8 lower (P < 0.001). If the ICSEA score was included as a covariate in the model, rather than the factor, high school type, there was a significant model effect (P = 0.014) with higher UMAT1 predicted for schools with a higher ICSEA score.

**Table 3:** UMAT and UMAT component scores, interview and interview component scores by student demographic characteristics after the revised selection processes (1999 to 2011).

	UMAT 1 Score	UMAT 2 Score	UMAT 3 Score	Total UMAT Score	Total Interview Score	Motivation/Commitment Score	Communication Skills Score
Gender							
Male (N = 657)	61.3 ± 0.3	55.6 ± 0.4	61.4 ± 0.4	178.3 ± 0.6	17.7 ± 0.1	2.88 ± 0.04	3.24 ± 0.04
Female (N = 780)	59.5 ± 0.3 ***	59.4 ± 0.3 ***	58.5 ± 0.3 ***	177.1 ± 0.6	18.7 ± 0.1 ***	3.06 ± 0.04 ***	3.52 ± 0.04 ***

High School							
Independent High School (N = 856)	60.8 ± 0.3	57.5 ± 0.3	60.0 ± 0.3	178.2 ± 0.5	18.2 ± 0.1	2.52 ± 0.03	2.89 ± 0.03
Public High School (N = 439)	59.2 ± 0.4 ***	57.1 ± 0.5	59.6 ± 0.4	175.6 ± 0.8 **	17.9 ± 0.2	2.43 ± 0.04	2.80 ± 0.04

Special Entry Pathways							
Special Entry (N = 276)	57.3 ± 0.5	56.3 ± 0.5	55.1 ± 0.5	168.9 ± 0.9	16.9 ± 0.2	2.29 ± 0.04	2.57 ± 0.05
No Special Entry (N = 1161)	61.0 ± 0.2 ***	58.0 ± 0.3 **	60.9 ± 0.3 ***	179.7 ± 0.4 ***	18.5 ± 0.1 ***	2.55 ± 0.02 ***	2.94 ± 0.02 ***

Region of Origin							
Oceania (N = 992)	60.8 ± 0.3 ***	57.6 ± 0.3	58.9 ± 0.3 *** ^###^	177.1 ± 0.5	18.3 ± 0.1 *	2.50 ± 0.02 *	2.88 ± 0.03 *
UK & Ireland (N = 52)	62.4 ± 1.2 *	58.3 ± 1.1	59.5 ± 1.2 ^#^	180.1 ± 2.2	18.4 ± 0.5	2.56 ± 0.10	3.06 ± 0.10 *
NE & SE Asia (N = 193)	58.3 ± 0.6	57.4 ± 0.8	62.8 ± 0.7	178.4 ± 1.2	17.5 ± 0.2	2.38 ± 0.06	2.70 ± 0.06
Southern Asia (N = 91)	59.1 ± 0.8	56.7 ± 0.8	64.3 ± 0.8	180.1 ± 1.4	18.3 ± 0.4	2.68 ± 0.08	2.89 ± 0.08
Other (N = 107)	59.1 ± 0.8.	59.9 ± 0.8.	59.6 ± 0.8. * ^##^	178.6 ± 1.5.	18.4 ± 0.4	2.52 ± 0.08	2.93 ± 0.08

P-values-independent sample T-test -* P < 0.05, ** P < 0.01, *** P < 0.001, ANOVA post hoc comparisons with Bonferroni correction-* P < 0.05, ** P < 0.01, *** P < 0.001 vs NE and SE Asia-^# ^P < 0.05, ^## ^P < 0.01, ^### ^P < 0.001 vs Southern Asia.

##### UMAT2 Score (Understanding people)

Univariate associations of demographic characteristics with UMAT2 score are listed in Table 3. The score for UMAT2 was higher in females compared to males. No significant univariate influences of region of origin or high school type were seen. Multivariate analysis (GLM) confirmed significant model effects on UMAT2 for gender (P < 0.001), region of origin (P = 0.033) and selection via special entry pathways (P = 0.015). Parameter estimates indicated that UMAT2 was 3.8 ± 0.5 higher in females compared to males (P < 0.001). There was no significant model effect from high school of origin. Compared to students born in Oceania, UMAT2 was 2.4 ± 0.9 higher in those from Other region of origin (P = 0.011). Compared to standard pathway entrants UMAT2 in those who entered via the rural special entry pathway was 2.1 ± 0.9 lower (P = 0.019) and in those who entered via the outer urban special entry pathway was 4.8 ± 2.1 lower (P = 0.022). If the ICSEA score was included as a covariate in the model, rather than the factor, high school type, there was a significant model effect (P = 0.041) with lower UMAT2 predicted for schools with a higher ICSEA score.

##### UMAT3 Score (Non-verbal reasoning)

Univariate associations of demographic characteristics with UMAT3 score are listed in Table 3. The score for UMAT3 was lower in females compared to males. It was substantially higher in students of NE and SE Asian origin and Southern Asian compared to both those born in Oceania or of UK & Irish origin and lower in students from rural public and rural independent high schools compared to those from public metropolitan or independent metropolitan schools. Multivariate analysis (GLM) confirmed significant model effects on UMAT3 for gender (P < 0.001), region of origin (P < 0.001), high school of origin (P = 0.007) and selection via special entry pathways (P < 0.001). Parameter estimates indicated that UMAT3 was 2.6 ± 0.5 higher in males compared to females (P < 0.001). Compared to students from independent metropolitan high schools, UMAT3 was 4.2 ± 1.2 lower in those from rural public high schools (P < 0.001). Compared to students born in Oceania, UMAT3 was 2.0 ± 0.8 higher in those from NE and SE Asia (P = 0.008) and 4.1 ± 1.1 higher in those from Southern Asia (P < 0.001). Compared to standard pathway entrants UMAT3 in those who entered via UWay was 5.6 ± 2.7 lower (P = 0.041) and via rural special entry was 3.6 ± 0.9 lower (P < 0.001). If the ICSEA score was included as a covariate in the model, rather than the factor, high school type, there was a significant model effect (P < 0.001) with higher UMAT3 predicted for schools with a higher ICSEA score.

##### Total UMAT Score

Univariate associations of demographic characteristics with the total UMAT score are listed in Table 3. The overall score was not significantly associated with either gender or region of origin. There was, however, a significant relationship to high school of origin. Multivariate analysis (GLM) confirmed a significant model effect on total UMAT score by selection via special entry pathways (P < 0.001) and high school of origin (P = 0.046) but no significant model effects for gender or region of origin. Parameter estimates indicated that compared to standard pathway entrants, total UMAT score in those who entered via UWay was 17.1 ± 4.4 lower (P < 0.001), via the rural special entry was 9.4 ± 1.4 lower (P < 0.001) and via the outer urban pathway was 7.1 ± 3.3 lower (P = 0.033). If the ICSEA score was included as a covariate in the model, rather than the factor, high school type, a significant model effect was seen (P = 0.011) with higher UMAT predicted for schools with a higher ICSEA score.

#### Interview Score

Univariate associations of demographic characteristics with the total interview score are listed in Table 3. The Interview score was higher in females compared to males but lower in students of NE and SE Asian origin compared to those in Oceania. Multivariate analysis (GLM) confirmed significant model effects on Interview for gender (P < 0.001), region of origin (P = 0.004) and selection via special entry pathways (P < 0.001) but not high school of origin or ICSEA score if it was included as a covariate in the model. Parameter estimates indicated that Interview score was 1.0 ± 0.2 higher in females compared to males (P < 0.001). Compared to students born in Oceania, Interview score was 1.1 ± 0.3 lower in those from NE and SE Asia (P = 0.008). Compared to standard pathway entrants Interview score in those who entered via rural special entry was 1.7 ± 0.3 lower (P < 0.001) and outer metropolitan special entry was 1.8 ± 0.8 lower (P = 0.021).

Univariate associations (Table 3) and multivariate analyses (data not shown) for the two consistently delivered components of the interview, a global communications skills score and a motivation/commitment score, showed identical outcomes to the total interview score

## Discussion

The introduction in 1999 of revised selection procedures, which added a structured interview and an aptitude test, rather than relying on academic performance alone, coincided with a subsequent substantial shift in the demographic make-up of high school students admitted to the undergraduate MBBS course at UWA. In part, this demographic shift reflected affirmative action policies for students of rural origin (initiated in 1993), outer urban origin (initiated in 2009) or those with other socio-educational disadvantage (UWay-since 1996). However despite this affirmative action, students after 1999 still tended to come predominantly from schools of higher socio-educational advantage, including those students admitted from public high schools. In addition, the proportion of females increased substantially while the proportion of students from a NE or SE Asian region of origin decreased dramatically. To what extent can the demographic shifts we have seen be linked to the introduction of UMAT and interview rather than the previous reliance on academic entry score alone?

### Gender

The change in gender balance in the cohort, with an increase in the number of females both applying for and selected into the course, was evident immediately in the first year after the introduction of the revised selection processes and continued thereafter. An increasing number of female applicants to medical school reflects an international trend which has prompted several explanations including changing social norms, economic factors, changing family composition and equality legislation [8]. In the UK the proportion of female applicants has stabilised at 56% [8] which corresponds closely with the 56.7% of female applicants in WA since 1998. It also closely approximates the proportion of females who sat the UMAT in 2010 (56.7%, total N = 16,458) [1]. Are male applicants experiencing disadvantage under the new admissions process? On the contrary, our analytic models indicate that an influence of higher interview score in females most likely determined this striking gender shift and brought the proportion of successfully selected females more into line with an increasing number of female applicants. Although there were highly significant differences in performance in the individual components of UMAT by gender, these were in contrasting directions and therefore there was no overall association of gender with the total UMAT score. Females performed better in UMAT2-Understanding People, while males performed better in UMAT1-Logical reasoning and problem solving-and UMAT3-Non-verbal reasoning. This reflects exactly the performance by gender reported for all those who sat the UMAT in 2010 with the UMAT1 score lower by 3.2, UMAT2 score higher by 2.4 and UMAT3 score lower by 2.7 in females compared to males [1]. Males entered with significantly higher academic entry score both before and after the revised selection process. This meant that when entry was based on academic performance alone, males were preferentially selected out of proportion to their relative number of applications. After the revised selection process, because the 3 components for entry were weighted equally, it is suggested that the higher academic entry score seen for males was now counterbalanced by the higher interview score for females, resulting in a proportionate selection of males and females relative to their respective number of applications. Of interest, at the University of Queensland where the interview was removed from the selection process in 2008, the reverse of our observation has been seen with the male:female ratio shifting from being in favour of females to being in favour of males (David Wilkinson-personal communication).

### High School of Origin and Socio-Educational Disadvantage

The proportion of students from public versus independent high schools entering the MBBS in WA has always been substantially lower than the relative proportion of students in public high schools. The selection of only 10% of students into our medical school from high schools with an ICSEA score less than or equal to the mean score of 1000 has parallels at an international level, with data from the UK indicating that only 15% of medical students come from the lowest socio-economic groups although they comprise 50% of society [8]. It also has parallels in our own region with a study from the University of Auckland in New Zealand, that calculated a decile score for socioeconomic status for each high school of origin as a surrogate for each student's socioeconomic background, and found that less than 25% of their students are being admitted from those schools in the bottom five socioeconomic deciles [9]. Although composition of medical schools has always been dominated by those from backgrounds of higher socio-educational advantage, it was anticipated that our revised selection processes would enhance equity in the selection of students with respect to different socio-economic backgrounds. However, it appears that this has largely not been realised. Even after adjustment for a background of a decreasing proportion of students enrolling in public vs independent high schools in WA, there was relatively little influence on the significantly higher proportion of students entering the MBBS from independent vs public high schools. Our data indicate that students from public high schools generally entered the course with lower academic entry scores, lower UMAT scores but similar interview scores. However, students entering from schools with a higher ICSEA score, whether public or independent, entered with a higher overall UMAT score and higher academic performance. This has meant that even within public metropolitan schools successful applicants have increasingly come from those public schools with a higher ICSEA score. This suggests that utilising UMAT and a structured interview as parameters for selection in addition to academic performance has not fulfilled the expectation of enhancing equity of access to the MBBS at UWA. The success of foundation programs in the UK that specifically select students with socio-educational disadvantage [10] suggests that the recent introduction of an Outer Metropolitan pathway at our medical school, which targets and recruits students from high schools with lower ICSEA scores into a preserved quota of places, may offer a more effective approach to redressing this longstanding comparative under-representation of students from disadvantaged socio-economic backgrounds.

### Region of Origin

There was a preponderance of students from NE or SE Asian region of origin in our medical school when the selection criterion was academic performance alone. Whether a more than ten-fold increase in representation by these students in the medical course relative to their background ethnic composition in the population represents a distortion in the selection process or whether medical schools should aspire to a diversity based on ethnic proportionality remain highly controversial issues. A high proportion of Asian students in medical schools is also seen in New Zealand but with 16% of the NZ population of Asian origin and growing, such overrepresentation is anticipated to be less marked into the future [11]. High application rates and successful entry of Asian students is also a feature in the UK [12], although more from a Southern Asian background, with that group constituting approximately 16% of applicants and 13% of those accepted, (against a background prevalence of approximately 4% of the UK population). Chinese constituted only 0.4% of the UK population but 2% of those both applying and accepted into medical schools [8]. The over-representation of Asian students in medical schools has been attributed to higher educational aspirations within their families as well as the prestige that their communities attach to a career in medicine [8]. In our medical school higher academic performance at high school has clearly been the major basis for such over-representation.

The large decrease in numbers of students from a NE or SE Asian region of origin following introduction of the revised selection processes was immediate, the temporal pattern of onset indicating a likely direct influence of the revised selection process. Any such influence appears to be linked more to performance in the interview rather than the UMAT. For the interview score (and two of its components-motivation/commitment score and communication skills score) performance was lower in students of NE or SE Asian region of origin compared to those from Oceania or UK and Ireland. However, while students of NE or SE Asian students scored lower in UMAT1 (Logical reasoning and problem solving) relative to students from either Oceania or UK and Ireland, there was a highly significant reversal of this trend for UMAT3 (Non-verbal reasoning) which resulted in there being no significant influence of region of origin on the total UMAT score.

The implication could be drawn that our interview process is discriminatory against students of NE or SE Asian region of origin, but this remains a simplistic explanation for their lower interview score. In the same interview students of Southern Asian origin have been performing at a comparable level to students born in Oceania or the UK and have increased their representation within the course since the introduction of the interview and UMAT. Cultural and/or language differences remain a more likely explanation for both differences in the interview score and UMAT component scores. In this respect, the Australian Council for Education Research in the 2010 annual UMAT report [1] observed that 33% of the 16,458 students who sat the test that year did not have English as their home language and that in at least 55% of those, the home language was of NE or SE Asian region of origin. When all subjects from a non-English speaking background were pooled, their performance in UMAT1 was lower by 3.5, in UMAT2 was lower by 5.6 and in UMAT3 higher by 0.7 [1]. In our smaller and highly selected sample we saw comparable outcomes for UMAT1 and UMAT3, but not UMAT2 for students of NE or SE Asian origin.

The continuing decline year on year in numbers of students from NE or SE Asian region of origin also reflects the likely further influence of the corresponding and incremental growth in students entering via the rural special entry pathway with 8 to 9 out of 10 applicants of rural origin having been born in Oceania.

### Rural Vs Metropolitan Origin

Affirmative action through special entry pathways into a high stakes course such as medicine will always be controversial. However, in 1988 despite the population outside the Perth metropolitan region being 38% of the total WA population (N = 455,200) [13] less than 3% of students selected for the MBBS course at that time were of rural origin. By 2010, the population outside of the Perth metropolitan area had grown to 597,400, 26% of the state's total [14] and therefore the establishment of a rural special entry pathway with a quota of 25% of all places in the MBBS appears to have been fully justifiable. This 25% quota has now been consistently achieved each year since 2006 and the previous substantial imbalance has now been fully redressed. Hopefully, consistently meeting the 25% quota into the future will achieve the further goal of graduating more medical practitioners who will choose to practise in otherwise severely under-serviced rural and remote regions throughout Australia.

### Study Limitations

Our analysis of the potential influences of UMAT and interview scores has been limited to the 35% of total applicants who were actually admitted to the course and would ideally have included an analysis of all applicants, not just those selected. This confinement of the analyses to those at the upper end of the distribution for UMAT and interview scores, however, is likely to have underestimated rather than overestimated the strength of the relationships identified in this retrospective cross-sectional analysis. The imputation of ICSEA scores calculated in 2010 to the high schools of students entering the course from 1985 to 2009 assumes stability in this index over time that may or may not be justifiable. Finally, although every attempt has been made to consider broader population changes that may have been responsible for our results, some unaccounted demographic or sociological trends may also have contributed.

## Conclusions

The introduction of a structured interview, but not an aptitude test, to the selection process at our medical school appears to have contributed to a restoration in gender balance with females now selected more in proportion to their background number of applications. The structured interview also appears to have exerted an influence on the region of origin of our students reducing a previous substantial over-representation by students of a NE or SE Asian region of origin. Together with special entry quotas for rural students, the revised selection processes have largely reversed the trends seen for these demographic factors when entry to the course relied on academic performance alone. At this point, however, there has been limited impact on the numbers of students recruited into the course from high schools with relative socio-educational disadvantage. We will monitor with eager anticipation future effects of the recent introduction of a special entry pathway to our course for students from outer urban high schools with clear socio-educational disadvantage. Medical schools utilising scores from structured interviews and aptitude tests as criteria for entry should be aware of the potential influence of these approaches to selection on the demographic composition of their student cohorts, especially with respect to gender and region of origin. Any changes to selection processes should be accompanied by careful evaluation of such possible effects on a prospective basis.

## Competing interests

The authors declare that they have no competing interests.

## Authors' contributions

IP contributed to the conception and design of the study, acquisition, analysis and interpretation of the data; and the initial drafting and final revision of the manuscript

AM contributed to the conception and design of the study, interpretation of the data; and final revision of the manuscript for important intellectual content

SC contributed to the interpretation of the data; and final revision of the manuscript for important intellectual content

BL contributed to the design of the study, interpretation of the data; and final revision of the manuscript for important intellectual content

All authors read and approved the final manuscript.

## Pre-publication history

The pre-publication history for this paper can be accessed here:

http://www.biomedcentral.com/1472-6920/11/97/prepub
